# Trigeminal neuralgia associated with ophthalmic surgery: a case series 

**DOI:** 10.1186/s13256-018-1795-8

**Published:** 2018-09-08

**Authors:** María del Pilar Lucena, Federico Furno Sola, Mitzy E. Torres Soriano, Fanny Andrea Gerster

**Affiliations:** 1 Ophthalmology Department, Sanatorio Mapaci. Grupo Laser Visión, Mariano Moreno 1397, CP 2000 Rosario, Santa Fe Argentina; 2 Retina Department at Centro de la Visión Gordon-Manavella and Clínica de Ojos “Dr. Carlos Ferroni”, Rosario, Santa Fe Argentina

**Keywords:** Trigeminal, Neuralgia, Retinal surgery, Pain

## Abstract

**Background:**

Trigeminal neuralgia is a rare cause of postoperative pain after ophthalmic surgery and has only been described twice in the international literature: one case of pain after vitrectomy with a crystalline lens extraction and another case of an unspecified surgery type. We report three cases of ophthalmic surgery-induced trigeminal neuralgia.

**Case presentation:**

Trigeminal neuralgia was diagnosed in three patients after ophthalmic surgery. Patient 1 was a 63-year-old Caucasian man with an epiretinal membrane and cataract in his left eye. Phacovitrectomy was performed. Patient 2 was a 38-year-old Caucasian man with a perforating wound in his right eye. Primary closure of the cornea with removal of the necrotic iris was performed. Patient 3 was a 52-year-old Caucasian man referred 15 days after suffering blunt trauma to his right eye. Vitrectomy was performed to remove the crystalline lens, which was luxated to the vitreous. On postoperative days, these three patients were admitted to an emergency ward due to sudden, shock-like, one-sided facial pain activated by numerous stimuli. After consultation with specialists from the anesthesia and neurology departments, a diagnosis of trigeminal neuralgia was made. This diagnosis was based on the presence of four of the nine clinical criteria described by the International Headache Society, which were met in these three cases.

**Conclusions:**

Trigeminal neuralgia is a rare cause of postoperative ophthalmic pain and is a challenging diagnosis for the ophthalmologist. It is necessary to consider the differential diagnosis of atypical pain after ophthalmic surgery.

## Background

Trigeminal neuralgia (TN) is a pain disorder affecting the fifth cranial nerve (trigeminal nerve) and is characterized by severe, paroxysmal, lancinating facial pain, especially in areas where some branches of the nerve are distributed. Pain bouts can last from a few days to a few months, with equal periods of remission.

Etiologically, essential TN is a neuropathic pain disorder, which is secondary to compression in the zone where the nerve enters the brainstem, and is often caused by tumors or, more frequently, blood vessels, cerebellar arteries, or venous drainage.

The vascular etiology has gained increasing importance because of the use of brain magnetic resonance imaging (MRI) and magnetic resonance angiography (MRA) to study these painful syndromes. Some anatomical studies of patients with essential TN have revealed that in 91% of cases, there is compression or contact of the blood vessels with the nerve root as it enters the brainstem. It has been proposed that vascular contact (usually arterial) and compression cause a loss of the myelin sheath from fibers, resulting in increased afferent activity of those fibers.

TN can be typical (or essential) or atypical; the first type refers to a painful syndrome that is limited to the distribution of a specific cranial pair or to some of its branches, and the latter develops secondary to an injury (tumor, post-therapeutic neuralgia, periodic migrainous neuralgia, central pain, or post-traumatic injury) [1].

Pain is triggered by a stimulation, such as wind blowing on one’s face, touching the face softly, moving the face, chewing, and talking, whereas heat or strong pressure on the face is not effective at triggering pain.

Diagnosis of TN is based on clinical signs and symptoms: a medical record with physical and neurological examinations. However, additional studies, such as X-rays, evoked potentials, computed tomography (CT), and MRI of the brain, focus on differentiating essential neuralgia from secondary neuralgia. MRA is the test of choice to assess vascular compression in the area of the fifth pair of cranial nerves in the brainstem.

There are several causes for pain after eye surgery, of which the most frequent causes are irritation of the ocular surface, high intraocular pressure (IOP), and swelling. TN is a rare cause of postoperative pain and has only been described twice in the international literature: one case of pain after vitrectomy with crystalline lens extraction [2] and another case of an unspecified surgery type [3].

We present a case series of three patients who presented with TN secondary to ophthalmologic surgery with general anesthesia, their treatment, and a literature review.

## Cases presentation

Three patients who presented with TN secondary to retinal surgery in August and September 2017 were included. These patients were diagnosed in the Sanatorio Mapaci and Ophthalmology Clinic, both of which are in Rosario city, Argentina. All three patients were male and ranged in age from 38 to 63 years.

One patient underwent a phacovitrectomy for epiretinal membrane and cataracts; another patient underwent surgery for primary closure of the cornea after a perforating injury; the final patient received vitrectomy for a crystalline lens extraction due to a dislocation in the vitreous cavity after blunt trauma. All procedures were performed in one single center by the same surgeon, and two different anesthesiologists participated in the procedures.

The three surgeries were performed under general anesthesia. None of the patients received an anesthetic nerve block.

### Case 1

A 63-year-old Caucasian man, a dentist, was referred by another professional with an epiretinal membrane and cataract in his left eye. His medical history revealed he had hypertension for the past 6 years under treatment. He had no relevant history of eye problems. His visual acuity test was 20/30 in his left eye. An ophthalmologic examination of his left eye revealed a nuclear cataract ++, epiretinal membrane with microfolds, and macular edema confirmed by a macular optical coherence tomography (OCT) scan. Phacovitrectomy was performed in his left eye under general anesthesia. On postoperative day 1, he did not experience pain and his visual acuity was 20/50. The findings included: a corneal edema, well-positioned intraocular lens, and Tyndall +. A fundoscopy showed an attached retina. On postoperative day 7, he did not experience pain and visual acuity was 20/20. The pseudophakia was unremarkable and an attached retina, without an epiretinal membrane, was observed on the fundoscopy. On postoperative day 20 he was admitted to an emergency ward due to severe eye pain that woke him up in the middle of the night. He described it as a severe, paroxysmal, lancinating facial pain and rated it as a 10/10 lasting 10 to 30 seconds. It radiated to the distribution of the first division of the right trigeminal nerve. He denied contralateral pain. On physical examination, he was neurologically intact. No family history of neurological problems was found. His visual acuity was 20/20. Pseudophakia and nasal choroidal detachment were observed. After consultation with specialists from the anesthesia and neurology departments, he was diagnosed as having TN with ophthalmic branch involvement. A complete blood count (CBC) test and liver function test were ordered and the results were unremarkable. Normal findings on both CT and MRI were reported. Treatment with tramadol and morphine was started. A good response to medical treatment was observed. He had some episodes of TN during the first 2 months. After 1 year of follow-up, he did not have any more episodes of TN.

### Case 2

A 38-year-old Caucasian man, a mechanic, presented at the Ophthalmology unit of Sanatorio Mapaci with a perforating wound in his right eye. He had no relevant past medical/family history or eye problems. Visual acuity was hand motion in his right eye. Slit lamp biomicroscopy showed a penetrating wound in the cornea between hours 3 and 7, anfractuous injury, iris prolapse, and grade IV hyphema. There was no visualization of the posterior segment structures. Primary closure of the cornea with removal of the necrotic iris was performed on the same day under general anesthesia without a nerve block. On postoperative day 1 he did not experience pain and his visual acuity was light projection; IOP was 6 mmHg. The corneal wound was sealed with seven stitches (10/00 nylon sutures), Seidel test was negative, and hyphema was grade IV. He made good progress during the following days. On postoperative day 6 he was admitted to an emergency room due to severe pain in the right side of his face activated by numerous facial stimuli. He described it as disabling lacerating pain and rated it as a 10/10. A neurological examination was unremarkable. On a visual acuity test, he was able to count fingers at 10 cm. Slit lamp biomicroscopy showed a sealed corneal wound and grade III hyphema; IOP was 16 mmHg. He was referred to a medical clinic and underwent anesthesiology, where he was diagnosed as having TN. Blood tests including CBC and liver function test were normal. Normal findings in both CT and MRI were reported.

Treatment with tramadol, pregabalin, and B12 complex was started; during the first 2 months of medication he lost 9 kg (20 pounds) from not eating for fear of exacerbating the pain. Chlorpromazine and carbamazepine were added and his course evolved with sporadic pain.

### Case 3

A 52-year-old Caucasian man, a construction worker, was referred 15 days after suffering a blunt trauma to his right eye. His visual acuity was no light perception. An ophthalmologic examination revealed a clear cornea, traumatic mydriasis, aphakia, and Tyndall +++. The findings included: intraocular pressure (IOP), 40 mmHg; a pale optic disc with well-defined edges; temporal retinal necrosis; and an intumescent crystalline lens at hour 6. Vitrectomy was performed to remove the crystalline lens. On postoperative day 1, he did not experience pain. The findings included: visual acuity; no light perception; traumatic mydriasis; aphakia; Tyndall ++; IOP, 12 mmHg; and temporal retinal necrosis. On postoperative day 7, he was admitted to an emergency room with severe and excruciating pain in the right side of his face, predominantly in his right eye. After consultation with specialists from the anesthesia and neurology departments, he was diagnosed as having TN, with ophthalmic branch involvement. Blood tests were unremarkable. Normal findings on both CT and MRI were reported. He reported that Valsalva’s maneuver triggered pain. Treatment with tramadol, pregabalin, and B12 complex was started. A good response to medical treatment was observed.

## Discussion

In this case series, we present three cases with TN after retinal surgery, a rare cause of postoperative ophthalmic pain and a diagnostic challenge.

Avicena described TN as “facial torture” in the tenth century. In 1756, TN was called “tic douloureux”, and since 1776, TN, as described by John Fothergill, has been described as a different clinical entity.

Rushton and Olafson set the classic clinical criteria for the diagnosis of TN that are currently used, which include severe paroxysmal pain limited to one or more divisions of the trigeminal nerve with periods of remission and unpredictable pain attacks, and no motor or sensory loss involving the nerve and trigger zones (points that trigger pain when stimulated) [4, 5].

TN usually occurs between the fifth and sixth decades of life and is usually unilateral. The mandibular branch is the most frequently affected, followed by the maxillary branch. The ophthalmic branch is the least frequently affected. Isolated involvement of the ophthalmic division occurs in only 2.8% of cases [6].

The third edition of the *International Classification of Headache Disorders* (ICHD-3) does not specify the diagnostic criteria for TN after ophthalmic surgery. We made diagnoses of TN based on the diagnostic criteria for painful post-traumatic trigeminal neuropathy. The criteria are:

1. Facial and/or oral pain in the distribution(s) of one or both trigeminal nerve(s) and fulfilment of criterion 3.
2. History of an identifiable traumatic event to the trigeminal nerve(s), with clinically evident positive (hyperalgesia, allodynia) and/or negative (hypesthesia, hypoalgesia) signs of trigeminal nerve dysfunction.
3. Evidence of causation, as demonstrated by both of the following:
  pain localized to the distribution(s) of the trigeminal nerve(s) affected by the traumatic event;
  pain developed < 6 months after the traumatic event.
4. Not better accounted for by another ICHD-3 diagnosis.

The diagnosis of TN in these cases was based on four criteria listed above, which were met in these three cases.

The differential diagnoses were headache and severe dry eye, as they share certain pathophysiological features within the trigeminal system [6]. An ophthalmologic examination did not reveal severe dry eye in any of the cases. In the three cases, headache was excluded after a physical examination, which revealed that the pain affected the distributed trigeminal nerve area and was triggered by movement of or contact with the face (unlike with headaches).

Only two cases of TN post-ophthalmic surgery [2, 3] and one case of TN post-retinal surgery [1] are reported in the literature. Neuropathic pain related to corneal diseases has also been described in the literature, especially because of a disrupted tear film or direct noxious stimulation of the corneal nerves [7].

Several theories have been developed to explain the etiology and pathophysiology of TN:

1. Theories related to demyelination at the ganglion level, which causes hypersensitivity of the trigeminal afferent branches, secondary to accumulation of sodium inside the neurons, which results in repeated electrical stimulation of the bulbar reticular formation, producing a conscious perception of neuralgia.
2. Jannetta’s theory, which suggests that there is a compression or malformation in the trigeminal dorsal root entry zone caused by malformations or vascular anatomical variations, the most important of which is the superior cerebellar artery, which is most frequently compressed (95% of cases).

These two theories can be dismissed since all three patients underwent an MRI and CT, which did not indicate compression or demyelination.

3. Fromm and colleagues’ [7] “epileptogenic theory” proposes that an irritation of the trigeminal nerve, in our case the ophthalmic surgeries performed under general anesthesia, but without retrobulbar blockage induces alterations in the segmental inhibitory systems (trigeminal sensory nuclei) and, therefore, causes an increase in the activity of these nuclei secondary to the activation of ectopic action potentials. The increased activity of the primary afferent fibers, together with the loss of the inhibitory mechanisms of the sensitive nuclei of the trigeminal nerve, lead to paroxysmal discharges in the interneurons of these nuclei in response to touch stimuli and, therefore, to pain crises.

However, we do not know the exact etiology and pathophysiology in these cases.

## Conclusions

In conclusion, TN is a rare cause of postoperative ophthalmic pain and is a challenging diagnosis for the ophthalmologist. It is necessary to consider this differential diagnosis of atypical pain after ophthalmic surgery.
